# Conditional Loss of MEF2C Expression in Osteoclasts Leads to a Sex-Specific Osteopenic Phenotype

**DOI:** 10.3390/ijms241612686

**Published:** 2023-08-11

**Authors:** Ravi Maisuria, Andrew Norton, Cynthia Shao, Elizabeth W. Bradley, Kim Mansky

**Affiliations:** 1 Department of Developmental and Surgical Sciences, School of Dentistry, University of Minnesota, Minneapolis, MN 55455, USA; maisu002@umn.edu (R.M.); aanorton2@wisc.edu (A.N.); 2 College of Biological Sciences, University of Minnesota, Minneapolis, MN 55455, USA; shaoc@umn.edu; 3 Department of Orthopedics, School of Medicine and Stem Cell Institute, University of Minnesota, Minneapolis, MN 55455, USA; ebradle1@umn.edu

**Keywords:** osteoclasts, MEF2, transcription factor, osteopenia

## Abstract

Myocyte enhancement factor 2C (MEF2C) is a transcription factor studied in the development of skeletal and smooth muscles. Bone resorption studies have exhibited that the reduced expression of MEF2C contributes to osteopetrosis and the dysregulation of pathological bone remodeling. Our current study aims to determine how MEF2C contributes to osteoclast differentiation and to analyze the skeletal phenotype of *Mef2c-*cKO mice (*Cfms-cre*; *Mef2c^fl/fl^*). qRT-PCR and Western blot demonstrated that *Mef2c* expression is highest during the early days of osteoclast differentiation. Osteoclast genes, including c*-Fos, c-Jun*, *Dc-stamp*, *Cathepsin K*, and *Nfatc1*, had a significant reduction in expression, along with a reduction in osteoclast size. Despite reduced CTX activity, female *Mef2c* cKO mice were osteopenic, with decreased bone formation as determined via a P1NP ELISA, and a reduced number of osteoblasts. There was no difference between male WT and *Mef2c-*cKO mice. Our results suggest that *Mef2c* is critical for osteoclastogenesis, and that its dysregulation leads to a sex-specific osteopenic phenotype.

## 1. Introduction

Despite the complexities that make up the skeleton, none would be possible without bone-forming osteoblasts and bone-resorbing osteoclasts. The balance between osteoblast and osteoclast activity guarantees mechanical strength and other critical functions, such as the protection of vital organs and serving as a reservoir for calcium and phosphate [1]. Osteoclasts originate from the myeloid/monocyte lineage and fuse to become multinucleated cells capable of resorbing bone [2]. The dysregulation of osteoclast activity leads to a pathology that is devastating to the host. For example, the dysfunction of osteoclast activity leads to osteopenia and osteoporosis, as bone resorption outpaces bone formation [3].

Various transcription factors and soluble factors exist that regulate osteoclasts and bone resorption [4,5,6]. Macrophage colony-stimulating factor (M-CSF) and receptor activator of NF-κB (Nuclear Factor-Kappa B) ligand (RANKL) are essential for osteoclast differentiation [7]. M-CSF is a growth factor that allows for the survival and differentiation of osteoclasts, while RANKL allows for osteoclast differentiation, fusion, and activation [7,8]. Multiple transcription factors play a role in regulating osteoclast differentiation; however, one transcription factor, nuclear factor of activated T-cells 1 (NFATc1), is essential for regulating osteoclast differentiation. Other transcription factors, such as microphthalmia associated transcription factor (MITF), PU.1, and activator protein 1 (AP-1), have been shown to form a complex with NFATc1 to regulate osteoclast gene expression [4,9].

Myocyte enhancement factor 2 (MEF2) is a robust family of transcription factors regulating cellular programs, including proliferation, differentiation, and morphogenesis, in various cell types, including muscle cells, osteoblasts, and osteoclasts [10,11,12,13]. The four genes that make up the MEF2 family, *Mef2a*, *b*, *c*, and *d,* have distinct functions determined by tissue types, cellular proteins, environmental cues, and the coexpression of other transcription factors [12,13]. Our lab’s previous work has investigated the roles of *Mef2a* and *Mef2d* in osteoclast differentiation. Osteoclasts derived from *Mef2a* or *Mef2d* knockout mice demonstrated impaired osteoclast differentiation. Interestingly, female *Mef2a* cKO mice are osteopetrotic, while male *Mef2a* cKO and male as well as female *Mef2d* cKO mice had no significant skeletal phenotype compared to their wild-type littermates [10]. Even more interestingly, in vivo male mice that were conditionally deleted for both *Mef2a* and *Mef2d* were osteopenic, even though in vitro osteoclast cultures from these mice were almost devoid of TRAP-positive cells [10]. These results suggest that the expression of MEF2A and D is required for M-CSF- and RANKL-stimulated osteoclast differentiation in vitro, but osteoclasts form in the absence of MEF2A and D in vivo via an unexplained RANKL-alternative pathway [10].

*Mef2c*’s role in multiple cell types has been well studied [12,13]. SNPs associated with the *Mef2c* locus are associated with osteoporosis and osteoporotic fractures [14,15,16]. *Mef2c* has been shown to regulate chondrocytes and osteoblast differentiation in mouse models [17]; however, its role in osteoclast differentiation has not been well studied. Recently, a study by Fujii et al. demonstrated that *Mef2c* is a positive regulator of osteoclast differentiation [11]. The conditional loss of *Mef2c* when using the *Mx1-Cre* model led to smaller osteoclasts and increased bone mass at six weeks of age. They further demonstrated that *Mef2c* promotes the expression of *c-Fos* and the induction of NFATc1. Lastly, mice deleted for *Mef2c* expression were resistant to arthritic bone erosion due to the inhibition of osteoclast differentiation [11].

In contrast to the Fujii et al. study, we demonstrate that female mice conditionally deleted for MEF2C in cells targeted by *C-fms cre,* which include macrophages and osteoclasts, are osteopenic compared to their wild-type (WT) and male littermates. Unexpectedly, we determined that in vitro osteoclast cultures and in vivo TRAP-stained bone sections demonstrate a reduction in osteoclast size. Our data suggest that MEF2C regulates osteoclast differentiation and may regulate cross-talk between macrophages/osteoclasts and osteoblasts.

## 2. Results

### 2.1. Three-Month Female C-KO Mice Display Osteopenia In Vivo

**Figure 1:**
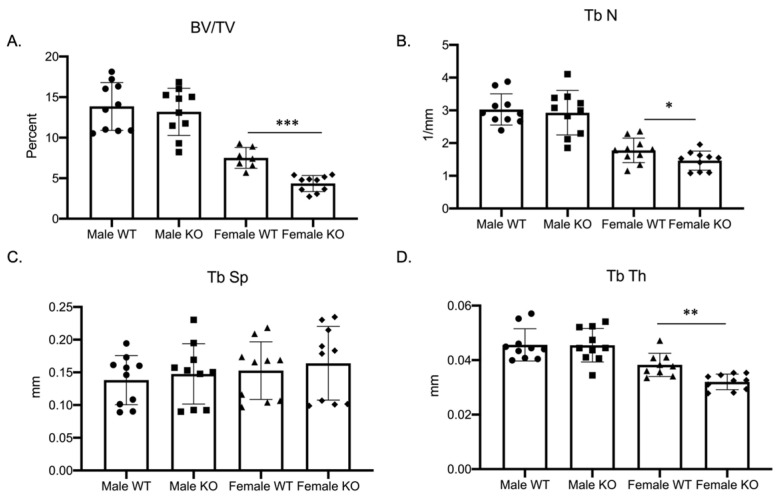
Female mice conditionally null for *Mef2c* are osteopenic. Data show trabecular micro-CT measurements for three-month-old male as well as female C-WT and C-KO mice. *n* = 7–10 mice per group for micro-CT measurements. (**A**) Ratio of bone volume to total volume, (**B**) trabecular number, (**C**) trabecular spacing, and (**D**) trabecular thickness. Bars show means ± SD. * *p* ≤ 0.05, ** *p* ≤ 0.01, and *** *p* ≤ 0.001 C-WT vs. C-KO.

Our previous study, examining the role of MEF2 transcription factors in regulating osteoclast differentiation, demonstrated a sex-specific skeletal phenotype in female mice conditionally deleted for the expression of MEF2A [10]. Due to the lack of studies examining the importance of *Mef2c* in osteoclast development, we investigated how the genetic ablation of *Mef2c* impacts osteoclast differentiation. To examine the role of *Mef2c* in osteoclast differentiation, we characterized the skeletal phenotype of *Mef2c^fl/fl^–C-fms Cre* male as well as female mice (Figure 1 and Supplementary Materials Figure S1, *n* = 10 wild-type and cKO male mice, 7 wild-type, and 10 cKO female mice). *Mef2c-flox/flox* (*Mef2c^fl/fl^*) mice (obtained from Dr. Eric Olson) were bred with *c-Fms-Cre* mice (*c-Fms-Cre^+/Tg^*; Jackson Laboratory) to obtain *Mef2c^fl/fl^–c-Fms-Cre^+/Tg^* (C-KO) and compared to *Mef2c^fl/fl^* (C-WT) mice. The phenotype for C-KO females was significantly different from female C-WT and from their male counterparts. Most noticeable was a significant decrease in the bone volume to total volume (BV/TV) ratio (Figure 1A, *p* ≤ 0.001), trabecular number (Figure 1B, *p* ≤ 0.05), and trabecular thickness (Figure 1D, *p* ≤ 0.01) in female C-KO versus C-WT bones. These significant decreases suggest an osteopenic phenotype in 3-month-old female C-KO mice.

### 2.2. Deletion of Mef2c Leads to Smaller In Vitro Osteoclasts

**Figure 2:**
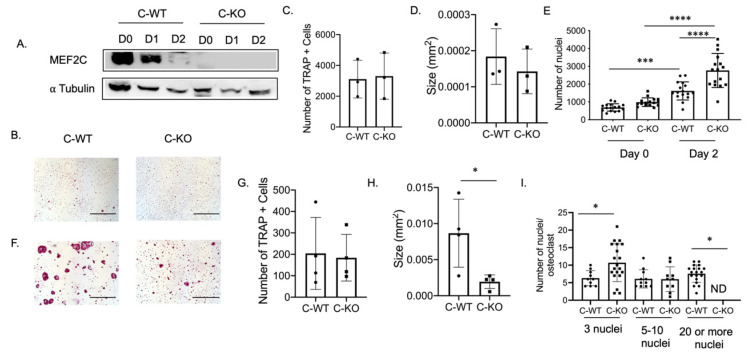
In vitro osteoclasts from female C-KO mice are smaller than C-WT osteoclasts. (**A**) Western blot of osteoclast lysates at day 0 (M-CSF only) or Day 1 or 2 (M-CSF and RANKL). (**B**) Representative TRAP images at Day 2. Scale bar = 0.5 mm (**C**) Average number and (**D**) size of TRAP+ cells. (**E**) Number of nuclei at Day 0 and Day 2. (**F**) Representative TRAP images at Day 4. Scale bar = 0.5 mm (**G**) Average number and (**H**) size of TRAP+ cells. (**I**) Number of nuclei/osteoclasts at Day 4. * *p* ≤ 0.05, *** *p* < 0.001, and **** *p* < 0.0001 C-WT vs C-KO.

To determine the expression pattern of *Mef2c* during osteoclast differentiation, we performed a Western blot using protein lysates extracted from bone marrow macrophages (BMM) from C-WT and C-KO mice. The protein lysates were cultured in M-CSF only (Day 0) or M-CSF and RANKL (Day 1 or 2)-containing media. MEF2C is highly expressed in lysates from C-WT cells on Day 0 and Day 1 (Figure 2A). MEF2C expression was not detectable in lysates from C-KO mice on any of the days during differentiation (Figure 2A).

**Figure 3:**
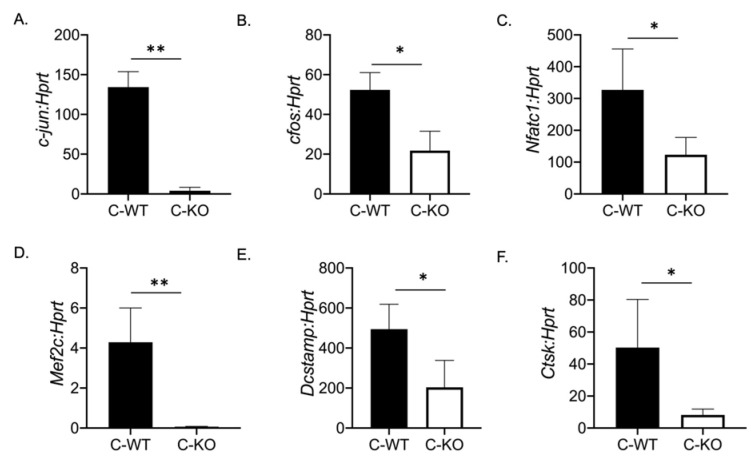
Osteoclasts from C-KO mice have reduced osteoclast gene expression. BMMs were isolated from C-WT as well as C-KO mice and cultured in M-CSF and RANKL for 2 days. RNA was isolated to measure gene expression via qRT-PCR. The relative expression of osteoclast marker genes normalized to WT controls against *Hprt* (**A**) *c-jun* ** *p* ≤ 0.01 C-WT vs. C-KO, (**B**) *c-Fos*, * *p* ≤ 0.05 C-WT vs. C-KO, (**C**) *Nfatc1*, * *p* ≤ 0.05 C-WT vs. C-KO, (**D**) *Mef2c*, ** *p* ≤ 0.01 C-WT vs. C-KO (**E**) *Dc-stamp* * *p* ≤ 0.05 C-WT vs. C-KO, and (**F**) *Cathepsin K* (*Ctsk*) * *p* ≤ 0.05 C-WT vs. C-KO,. Bars show means ± SD.

To determine if a change in osteoclast number was responsible for the osteopenic skeletal phenotype we observed via micro-CT, we cultured BMMs from female C-WT and C-KO mice. We did not detect any significant difference in the number of TRAP-positive cells on Day 2 or Day 4 of RANKL treatment, indicating that the number of osteoclasts did not differ between WT and C-KO mice (Figure 2B,C,F,G). Similarly, we did not detect any decrease in cell number via the DAPI staining of cell cultures from C-KO mice at either Day 0 or Day 2 compared to C-WT mice (Figure 2E, *p* ≤ 0.001 and *p* ≤ 0.0001). However, when comparing the size of osteoclasts, C-KO osteoclasts were smaller than C-WT osteoclasts after 4 days of RANKL treatment regardless of whether the cells were isolated from male or female mice (Figure 2H and Supplementary Materials Figure S2A, *p* ≤ 0.05). The number of osteoclasts with three nuclei were significantly higher in the TRAP-positive cells from the C-KO mice; however, there were no detectable cells with 20 or more nuclei as seen in the cultures from the C-WT mice (Figure 2I, *p* ≤ 0.05). Lastly, we also determined the expression of genes involved in osteoclast differentiation. We measured the significant downregulation of all of the genes tested, suggesting that the loss of MEF2C expression may regulate osteoclast differentiation at early stages through the expression of *c-fos* and *c-jun* (Figure 3).

### 2.3. In Vivo Osteoclasts are Decreased in Female C-KO Mice

**Figure 4:**
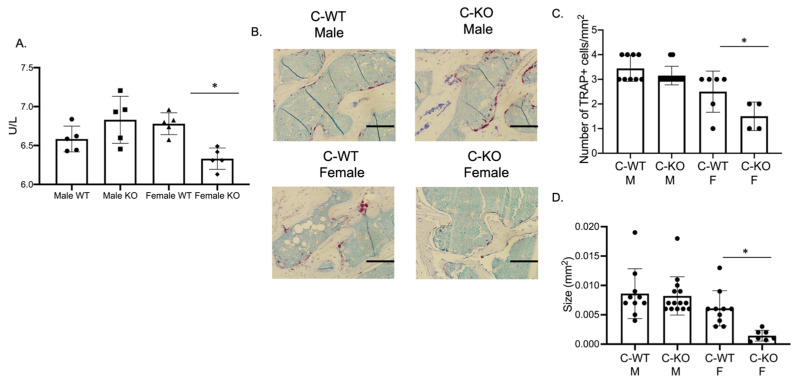
In vivo osteoclasts are decreased in female C-KO mice. Three-month-old C-WT and C-KO mice serum bone biomarkers were analyzed via an ELISA. (**A**) TRAP ELISA * *p* ≤ 0.05 C-WT vs. C-KO. (**B**) Representative images of TRAP-stained sections of trabecular bone. Scale bar = 10 mm (**C**) Number and (**D**) size of in vivo osteoclasts. Bars show means ± SD. * *p* ≤ 0.05 C-WT vs. C-KO.

**Figure 5:**
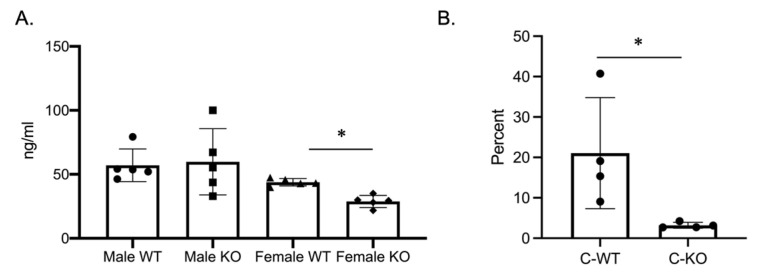
Osteoclasts isolated from MEF2CcKO mice have reduced activity. (**A**) Three-month-old C-WT and C-KO mice CTX activity was analyzed via an ELISA. * *p* ≤ 0.05 C-WT vs. C-KO (**B**) Percent demineralization of calcium-coated plates via osteoclasts from C-WT and C-KO mice. * *p* ≤ 0.05 C-WT vs. C-KO.

Our in vitro phenotype of smaller osteoclasts did not explain the in vivo osteopenic skeletal phenotype that we observed for our female C-KO mice. To determine in vivo osteoclast number and activity, we performed tartrate-resistant acid phosphatase (TRAP) and type 1 collagen (CTX) ELISAs from the serum of our male as well as female C-WT and C-KO mice. Female C-KO mice had reductions in serum markers for osteoclast number (Figure 4A, *p* ≤ 0.05) and activity (Figure 5A, *p* ≤ 0.05). We also measured a decrease in the ability of osteoclasts from C-KO mice to demineralize calcium-phosphate-coated plates (Figure 5B, *p* ≤ 0.05). The number and size of osteoclasts per bone surface via histology were also reduced (Figure 4B–D, *p* ≤ 0.05). These in vivo data agreed with our in vitro data showing that the loss of MEF2C expression leads to impaired osteoclast differentiation.

### 2.4. Female C-KO Mice Have Decreased Bone Formation

**Figure 6:**
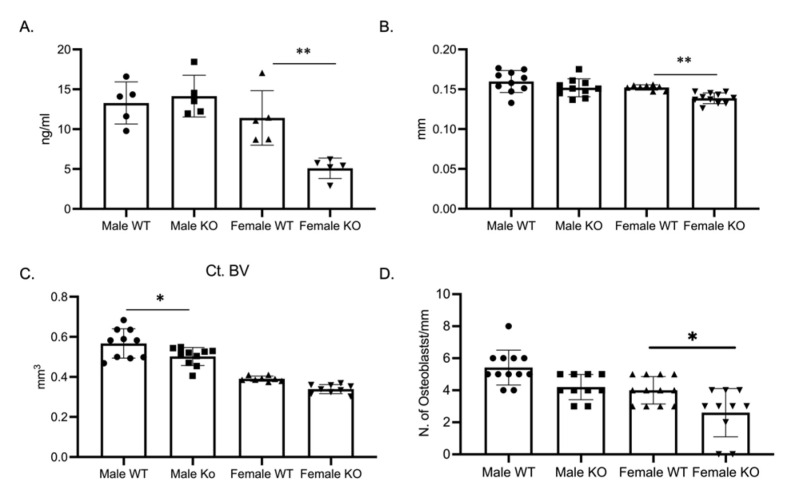
Bone formation and osteoblast numbers are decreased in female C-KO mice. Three-month-old C-WT and C-KO mice were analyzed for cortical bone parameters via micro-CT. (**A**) ELISA analysis of P1NP as a marker of bone formation. ** *p* ≤ 0.01 C-WT vs. C-KO. (**B**) Comparison of cortical thickness. ** *p* ≤ 0.01 C-WT vs. C-KO. (**C**) Comparison of cortical bone volume. * *p* ≤ 0.05 C-WT vs. C-KO (**D**) Number of osteoblasts per mm of bone. * *p* ≤ 0.05 C-WT vs. C-KO.

As the osteoclast phenotype did not explain the skeletal phenotype, we determined bone formation through a P1NP ELISA. P1NP, a marker of bone formation, was significantly decreased in female C-KO mice (Figure 6A). With the decrease in P1NP, we measured cortical thickness and cortical bone volume via micro-CT. Micro-CT analysis revealed a significant decrease in cross-sectional thickness in female C-KO mice but not in cortical bone volume (Figure 6B,C, Supplementary Materials Figure S3). Numbers of osteoblasts were determined via analyzing hematoxylin and eosin (H and E)-stained sections of femurs from C-WT and C-KO mice. There was a reduction in the number of osteoblasts in femurs from female C-KO mice compared to female C-WT mice (Figure 6D, Supplementary Materials Figures S4 and S5).

## 3. Discussion

*Mef2c* and the MEF2 family have been extensively studied in myocytes and neural cells. Additionally, MEF2 factors are evolutionarily conserved and function collaboratively through various transcription factors and networks [12,13]. Few studies have investigated the implications of *Mef2c* and bone resorption, despite their significance in bone healing and bone mineral density [14]. Here, we demonstrate that female *Mef2c* cKO mice (*Cfms-cre*; *Mef2c^fl/fl^*) are osteopenic compared to their wild-type (WT) littermates.

We begin by characterizing the skeletal phenotype of female and male *Mef2c*-cKO mice. The skeletal phenotype for C-KO females was significantly different from that of female C-WT and their male counterparts (Supplementary Materials Figure S5). A previous study examining C-KO mice demonstrated an osteopetrotic phenotype of male C-KO mice; however, in our study, the C-KO females were osteopenic compared to their WT littermates [11]. Differences between the phenotype measured in the two studies may be due to the differences in the Cre-expressing mice used (*Mx1-Cre* for [11], and *Cfms-Cre* in our study). *Mx1-Cre* is an inducible system and targets hemopoietic cells, while *C-fms-Cre* targets macrophages, osteoclasts, and dendritic cells.

Surprisingly, we did not measure a significant change in the number but instead in the size of osteoclasts from female C-WT and C-KO mice, with the size of osteoclasts being significantly different on Day 4. We were unable to identify osteoclasts with 20 or more nuclei in cultures from C-KO mice. These data, along with no significant change in the number of TRAP-positive cells, suggests that C-KO osteoclasts are not changed in their ability to form TRAP-positive cells but instead in their ability to fuse into large multinuclear cells. Furthermore, serum amounts for tartrate-resistant acid phosphatase (TRAP) and type 1 collagen (CTX), used to assess osteoclast activity, were significantly lower. Given this in vitro phenotype, we would have anticipated having an osteopetrotic skeletal phenotype; however, *P1NP,* the N-terminal propeptide of type 1 collagen and a marker of bone formation as well as the number of osteoblasts per mm, was reduced in our female C-KO mice compared to female C-WT mice. Our data suggest that the osteopenic skeletal phenotype we determined in the female C-KO mice may be due to a decrease in bone formation.

Given the expression pattern of MEF2C during osteoclast differentiation, we hypothesize that changes in MEF2C expression may change secreted factors from either macrophages or preosteoclasts that regulate bone formation [18,19,20,21,22,23]. Changes in secreted factors by macrophages or osteoclasts in C-KO mice may explain the osteopenic phenotype. We measure changes in both osteoclast and osteoblast activity (CTX and a P1NP ELISA). The osteopenic phenotype of the female MEF2CcKO indicates that while there is a decrease in both bone formation and resorption, based on the skeletal phenotype, the decrease in bone formation drives the bone phenotype. Based on our previous studies with the *cFms-cre* mouse line (MEF2A and HDAC7), we hypothesize that the skeletal phenotype of MEF2CcKO mice is not due to loss of MEF2C in osteoblasts but due to a change in MEF2C expression in one of the cells targeted by the *Cre* mouse line. We were able to count the number of osteoblasts/mm in H and E-stained bone sections; however, to more conclusively determine if changes in osteoblast number are the reason for the osteopenic phenotype, undecalcified bone sections should be used to more rigorously quantitate osteoblast numbers.

Factors such as oncostatin M (OSM) and low-density lipoprotein receptor related protein one (LPR1) have been shown to be expressed by macrophages to recruit and activate osteoblasts [18,24]. The role of macrophages in regulating bone formation has been well studied, especially during endochondral and intramembranous bone healing, demonstrating that macrophages are able to regulate bone marrow mesenchymal stem cells (BMSCs), osteoblasts, and osteocyte activity [25]. In fracture healing, inflammatory macrophages (M1) initiate events that contribute to tissue homeostasis through phagocytosing invading microorganisms, amplifying the inflammation response, and recruiting additional immune cells. As the tissue insult is cleared, M2 macrophages help with repair by secreting anti-inflammatory factors, recruiting osteoblast progenitors, and producing growth factors. Osteal macrophages, or osteomacs, are a specialized group of macrophages that support bone homeostasis and regeneration [19]. Osteomacs have been shown to express the cell surface receptor CD169. The loss of CD169-expressing cells in a mouse model resulted in a reduction in osteoblasts [26]; however, it is not evident from our current study if the loss of MEF2C expression affects the ability of macrophages to support bone formation.

The role of osteoclasts in regulating osteoblast activity has also been well studied. Osteoclasts have been shown to regulate osteoblast activity through soluble and membrane factors [27]. Recent studies with human biopsies treated with a single dose of Densomab, a humanized monoclonal antibody against RANKL, suggest that osteoclasts’ resorption exposes proteins in the extracellular matrix (ECM) that regulate bone formation [28]. Understanding the mechanism through which the expression of MEF2C in macrophages, preosteoclasts, and osteoclasts regulates bone formation is an interesting question but beyond the scope of this study.

Similar to our study with MEF2A, only female C-KO mice had a significant skeletal phenotype compared to their C-WT and male littermates [10]. Multiple studies have determined that estrogen is the primary hormone regulator of the skeleton in both men and women [29,30,31]. In previous studies looking at the role of MEF2 and class IIA HDACs in cardiac cells where estrogen has a cardioprotective effect, estrogen receptor alpha was shown to be a direct target of MEF2; however, they did not specify which MEF2 factor [32]. We observed an osteopenic phenotype in our C-KO female, but not in our male, mice. This study, as well as our previous study on MEF2A and MEF2D in osteoclasts, suggests that, in osteoclasts, the expression and/or activity of the estrogen receptor may be regulated by MEF2. Further studies will be needed to elucidate the potential role of the MEF2 family of transcription factors and estrogen receptor expression in osteoclast precursors. 

In summary, female *Mef2c* cKO mice (*Cfms-cre*; *Mef2c^fl/fl^*) display an osteopenic phenotype compared to their wild-type (WT) littermates. As exemplified through our in vitro and in vivo data, traditional bone resorption markers between *Mef2c* cKO mice and WT littermates during osteoclast differentiation are significantly downregulated. Our study further suggests that MEF2C may regulate macrophage/preosteoclast and osteoblast coupling.

## 4. Material and Methods

### 4.1. In Vitro Analysis

#### 4.1.1. Primary Osteoclast Cell Culture

BMMs were harvested from the femora and tibiae of both male and female mice at 3 months of age and differentiated into osteoclasts, as has been performed previously, with minor modifications [10]. Beginning two days after replating BMMs (referred to as Day 0), BMMs were fed 1.5% CMG 14-12 supernatant (Dr. Sunao Takeshita, Nagoya City University, Nagoya, Japan) containing M-CSF and 10 ng/mL RANKL (R&D Systems, Minneapolis, MN, USA) every 48 h (on Days 0, 2, and 4) until the desired experimental endpoint.

#### 4.1.2. DAPI Staining

Cells were cultured to the desired time point, fixed in 4% paraformaldehyde for 20 min at 4 °C, and then washed in PBS. Nuclei were stained using DAPI for five minutes at room temperature. The DAPI stain was then replaced with PBS. Cells were imaged using cellSens software (Olympus, version 1.6) and analyzed using NIH ImageJ (https://imagej.nih.gov/ij/download.html, accessed on 1 April 2023).

#### 4.1.3. Tartrate-Resistant Acid Phosphatase (TRAP) Staining

Cells were cultured to the desired time point, fixed in 4% paraformaldehyde for 20 min at 4 °C, and then washed in PBS. TRAP activity was stained for via the use of Naphthol AS-MX phosphate and Fast Violet LB salt. Cells were imaged using cellSens software (Olympus, version 1.6) and analyzed via the use of NIH ImageJ (https://imagej.nih.gov/ij/download.html, accessed on 1 April 2023).

#### 4.1.4. RNA Extraction and Analysis

RNA was isolated from cells plated in triplicate via the use of Trizol reagent (Ambion, Life Technologies, Austin, TX, USA) and quantified using UV spectroscopy. To synthesize cDNA with iScript cDNA synthesis kit (Bio-Rad, Hercules, CA, USA) as per the manufacturer’s protocol, 1 μg of RNA was used. Quantitative real-time PCR (RT-qPCR) was performed via the use of a MyiQ Single Color Real-Time PCR Detection System (Bio-Rad, Hercules, CA, USA). Each 20 μL reaction contained 1 ul of cDNA, 10 μL of iTaq Universal SYBR Green Supermix, and 25 μM of forward and reverse primers. The PCR conditions were as follows: 95 °C for 3 min, 40 cycles of 94 °C for 15 s, 58 °C for 30 s, and 72 °C for 30 s, followed by a melting curve analysis (95 °C for 5 s, 65 °C for 5 s, and then 65 °C to 95 °C with a 0.5 °C increase every 5 s). Experimental genes were normalized to *Hprt*. All measurements were performed in triplicate and analyzed through the ΔΔCT method. A list of primers is included in Table 1.

#### 4.1.5. Immunoblotting

Protein cell lysates were harvested from primary osteoclasts in a modified RIPA buffer (50 mM Tris pH 7.4, 150 mM NaCl, 1% IGEPAL, 0.25% sodium deoxycholate, and 1 mM EDTA) supplemented with a Halt Protease & Phosphatase Inhibitor Cocktail (Thermo Scientific, Waltham, MA, USA). Lysates were cleared via centrifugation. Proteins were resolved through SDS-PAGE and transferred to a PVDF membrane (Millipore, Burlington, MA USA). Blots were blocked in TBS/0.1% Tween-20 (TBST) plus 5% nonfat dry milk and incubated at 4 °C overnight with primary antibodies diluted in TBST plus 5% bovine serum albumin. The primary antibodies used are included in Table 2. The next day, blots were washed with TBST and incubated for 1 h at room temperature with horseradish-peroxidase conjugated secondary antibodies diluted in TBST plus 5% nonfat dry milk. The secondary antibodies used were from G.E. Health Systems: Amersham ECL anti-rabbit (NA-934) at 1:6000. Blots were washed in TBST before antibody binding was detected using a Western blotting detection kit Western Bright Quantum (Advansta, San Jose, CA USA) and the ChemiDoc Imaging System (Bio-Rad, Hercules, CA USA). Alpha tubulin was used as a loading control for all of the blots.

### 4.2. In Vivo Analysis

#### 4.2.1. Ethics

The use and care of the mice were reviewed and approved by the University of Minnesota Institutional Animal Care and Use Committee, IACUC protocol number 2104-39006A. Euthanasia was performed via CO_2_ inhalation.

#### 4.2.2. Mice

*Mef2c^fl/fl^* mice in a C57Bl/6 background were obtained from Dr. Eric Olson (University of Texas-Southwestern). *Mef2c^fl/fl^* mice were bred with *cFms-Cre^+/Tg^* mice (Jackson Laboratory) in an FVB/NJ background, and their progeny were crossed for individual analyses to obtain *Mef2c^fl/fl^*; *cFms-Cre^+/Tg^* (C-KO) and *Mef2c^fl/fl^* (C-WT). *Mef2c* depletion was verified via qRT-PCR and Western blot. 

#### 4.2.3. Sample Harvest

Three-month-old mice were used for in vivo experiments. Upon euthanasia, whole blood from the heart was drawn and centrifuged to obtain serum, which was immediately frozen until use. Femora and tibiae were removed and de-fleshed. For each mouse, the right femur was immediately stored in PBS and frozen without fixation for micro-CT. The left tibia was fixed in Z-fix (Anatech LTD, Battle Creek, MI, USA) and decalcified in 10% EDTA (pH of 7.4) for paraffin-embedded sectioning and histological staining. The left femur was used to harvest BMMs for in vitro culturing. Lastly, a piece of the tail was cut to verify the genotype of each mouse.

#### 4.2.4. Micro-CT Analysis

Frozen right femora were equilibrated to room temperature and scanned in PBS with a 1 mm aluminum filter via an XT H 225 micro-computed tomography machine (Nikon Metrology Inc., Brighton, MI, USA) at an isotropic voxel size of 7.4 µm. The scan settings were 120 kV, 61 µA, 720 projections, 2 frames per projection, and an integration time of 708 milliseconds. CT Pro 3D (Nikon Metrology, Inc., Brighton, MI, USA) was used to make 3D reconstruction volumes for each scan. VGStudio MAX 3.2 (Volume Graphics GmbH, Heidelberg, Germany) was used to convert 3D reconstruction volumes into bitmap datasets for each scan. A morphometric analysis was completed with a SkyScan CT-Analyser (CTAn, Bruker micro-CT, Billerica, MA, USA) following Bruker’s instructions and reported guidelines for the field. The region of interest for a trabecular bone analysis in the distal metaphysis started 0.5 mm proximal to the growth plate and extended 1.5 mm proximally towards the diaphysis. The region for a cortical bone analysis was a 0.5 mm region at the mid-diaphysis. Automated contouring was used to determine the region of interest boundaries for both trabecular and cortical bone, with manual editing as needed. Global thresholding was used to segment bone from surrounding tissue for 3D trabecular and 2D cortical analyses. One threshold value was used for all cortical analyses, and a different threshold value was used for all trabecular analyses. CT-Volume (Bruker micro-CT, Belgium) was used to create all 3D models from bitmaps corresponding to the cortical and trabecular regions analyzed; however, only a 1 mm region of the most distal trabecular selection was used to create a model.

#### 4.2.5. ELISAs

The detection of mouse CTX (IDS), TRAcP5b (IDS), and P1NP (IDS) in serum via ELISAs was carried out by following the manufacturer’s protocols (Immunodiagnostic Systems, Mountain Lakes, NJ, USA). Each sample was read in duplicate and averaged before analysis.

#### 4.2.6. Staining of Paraffin-Embedded Sections

Decalcified bone sections were deparaffinized in xylene, rehydrated through an ethanol gradient, and stained for TRAP at 37 °C for 1 h, as described above. Sections were then counterstained with methyl green for 15 s, cover slipped using Permount mounting media (Electron Microscopy Sciences, Hatfield, PA USA), and allowed to rest for 24 h before imaging.

### 4.3. Statistical Testing

The data presented in graphs for in vitro experiments represent an average of at least three independent experiments performed with bone marrow cells from mice cultured at different times. *n* = 4 for all in vitro TRAP-stained experiments. *n* = 3 for all RT-qPCR experiments. In vivo data represent all of the samples harvested for that specific experiment graphed together. *n* = 8–14 for all groups in micro-CT experiments. *n* = 6–11 per group for ELISAs and *n* = 5–9 for histology. An unpaired Student’s T test was used when comparing only two groups. A one-way ANOVA with Tukey’s multiple-comparisons test was used when comparing three or more groups. All statistical testing was performed in GraphPad Prism 8.

## 5. Conclusions

This study and our previous study suggest that MEF2A and C transcription factors regulate osteoclast differentiation in a sex-specific manner. Future studies will need to understand the relationship between estrogen and MEF2A and C in the regulation of osteoclast differentiation.

## Figures and Tables

**Table 1:** qRT-PCR primer sequences.

Primer Name	5′-3′ Sequence
Cathepsin K (CTSK) qPCR F	AGG GAA GCA AGC ACT GGA TA
Cathepsin K (CTSK) qPCR R	GCT GGC TGG AAT CAC ATC TT
C-Fos qPCR F	CCA AGC GGA GAC AGA TCA ACT T
C-Fos qPCR R	TCC AGT TTT TCC TTC TCT TTC AGC AGA
C-Jun qPCR F	TCC CCT ATC GAC ATG GAG TC
C-Jun qPCR R	TGA GTT GGC ACC CAC TGT TA
DC Stamp qPCR F	CAG ACT CCC AAA TGC TGG AT
DC Stamp qPCR R	CTT GTG GAG GAA CCT AAG CG
HPRT qPCR F	GAG GAG TCC TGT TGA TGT TGC CAG
HPRT qPCR R	GGC TGG CCT ATA GGC TCA TAG TGC
NFATc1 qPCR F	TCA TCC TGT CCA ACA CCA AA
NFATc1 qPCR R	TCA CCC TGG TGT TCT TCC TC
*Mef2c* qPCR F	AAG AAA CAC GGG GAC TAT GG
*Mef2c* qPCR R	ACA GCT TGT TGG TGC TGT TG

**Table 2:** Antibodies used for immunoblotting.

Target	Host Species	Vendor	Catalog Number	Lot Number
*Mef2c*	Rabbit	Abcam	AB211493	17163305
Alpha Tubulin	Rabbit	Cell Signaling	2144S	7

## Data Availability

Data will be available upon request.

## Supplementary Materials

The supporting information can be downloaded at: https://www.mdpi.com/article/10.3390/ijms241612686/s1.
