# Supplementary material for: Conditional Loss of MEF2C Expression in Osteoclasts Leads to a Sex-Specific Osteopenic Phenotype
Source: Int J Mol Sci. 2023 Aug 11;24(16):12686. doi: 10.3390/ijms241612686 (PMC10454686; doi:10.3390/ijms241612686)
Supplement: Supplementary file 1 [file ijms-24-12686-s001.zip › ijms-2387456-supplementary.pdf]

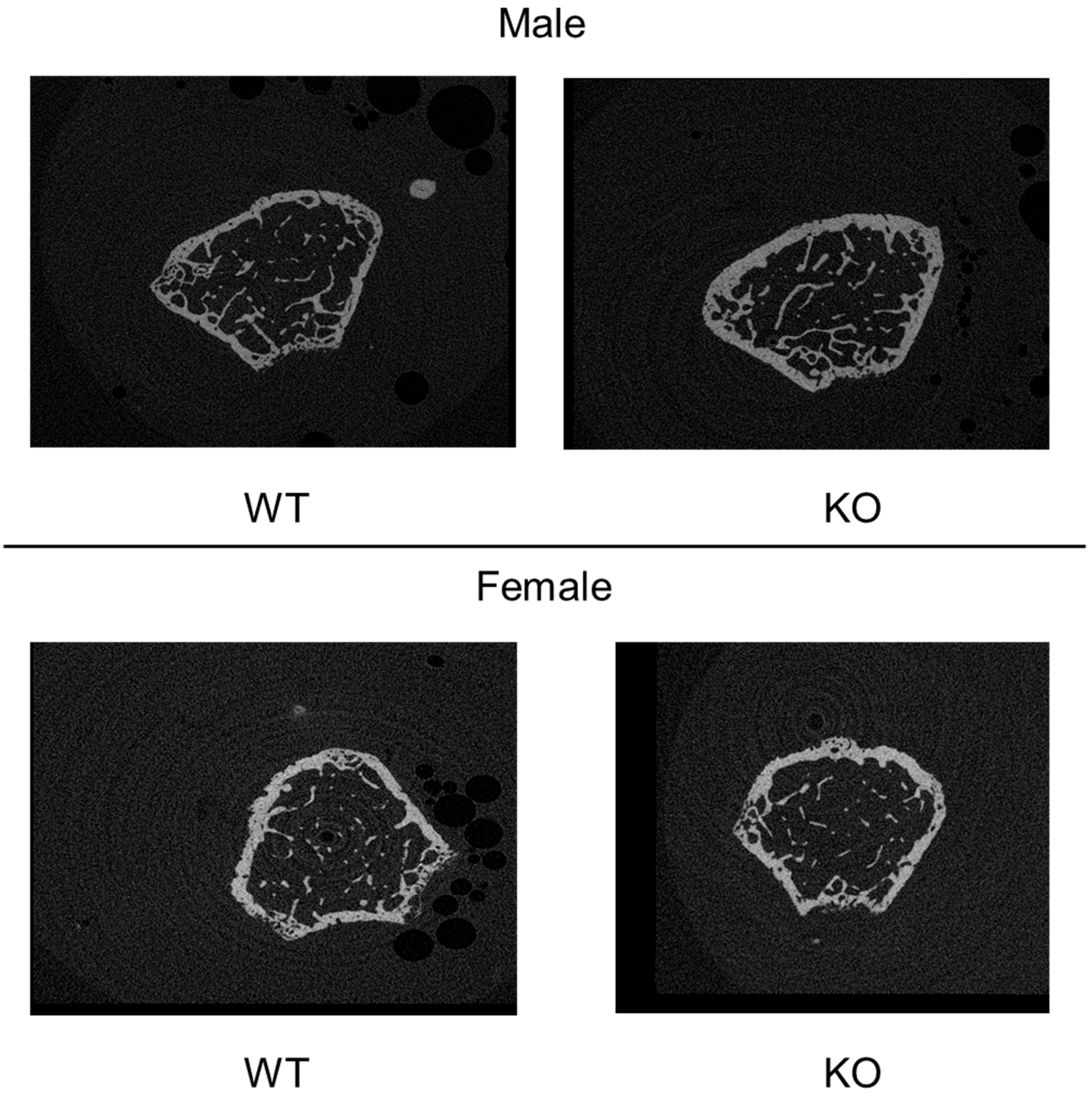

Figure S1. Representative micro-CT trabecular bone images of male as well as female C-WT and C-KO mice.

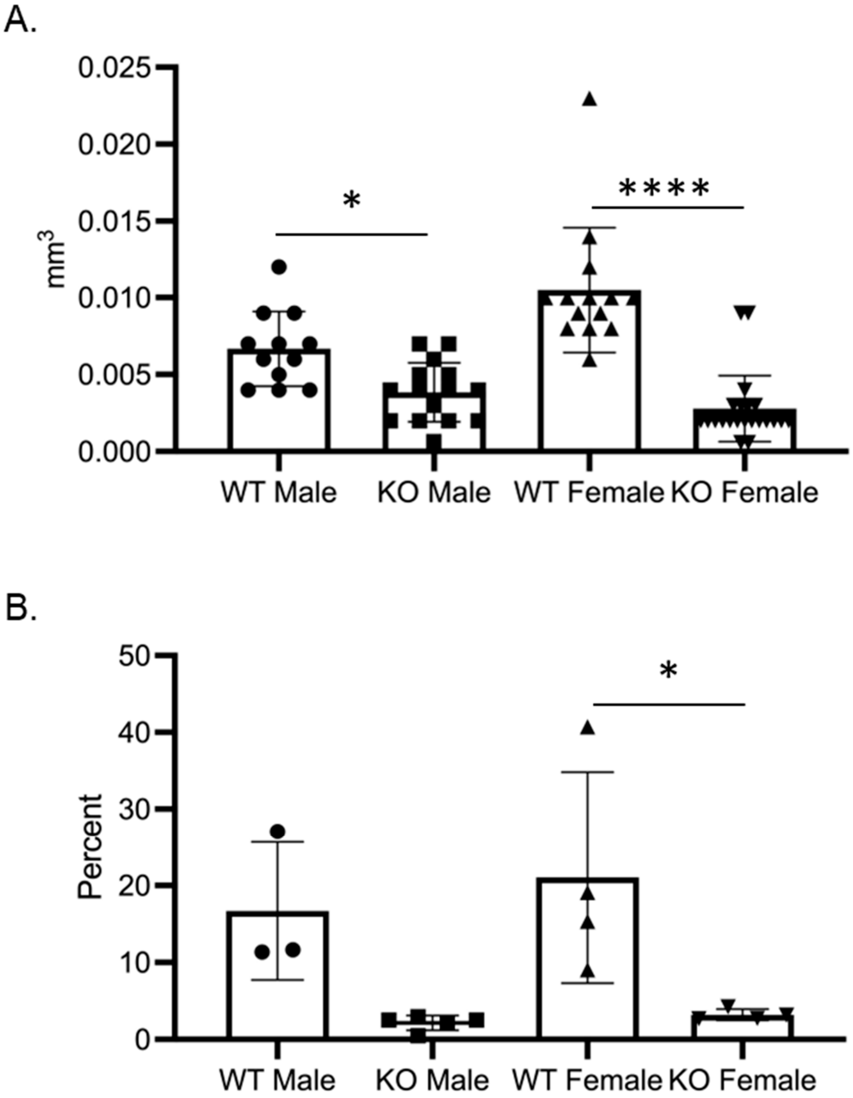

Figure S2. (A) Size of TRAP+ cell after 4 days in M-CSF and RANKL from male as well as female C-WT and C-KO mice. (B) Percent area demineralized by TRAP+ cells on calcium-coated plates by male as well as female C-WT and C-KO mice. *P $\leq$ 0.05 and ****P $\leq$ 0.0001 C-WT vs. C-KO.

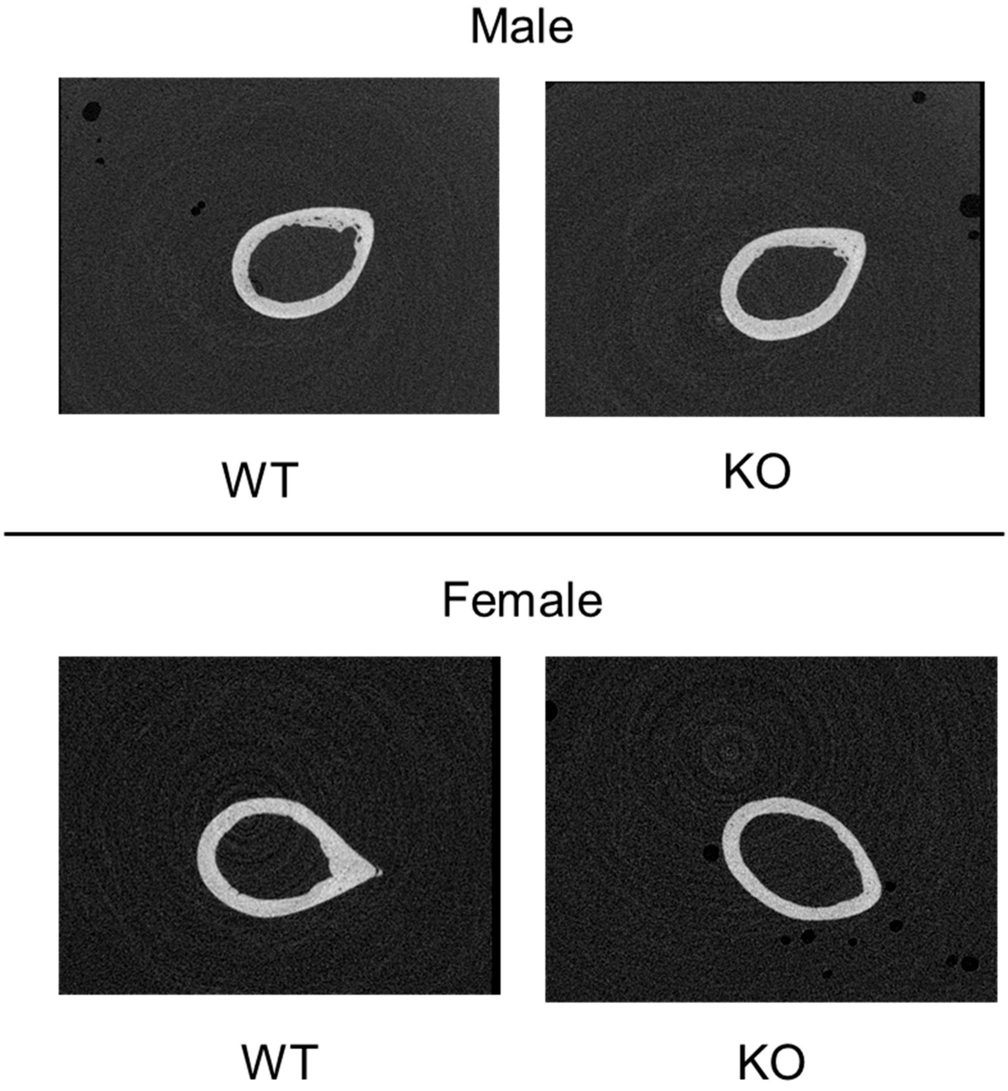

Figure S3. Representative micro-CT cortical bone images of male as well as female C-WT and C-KO mice.

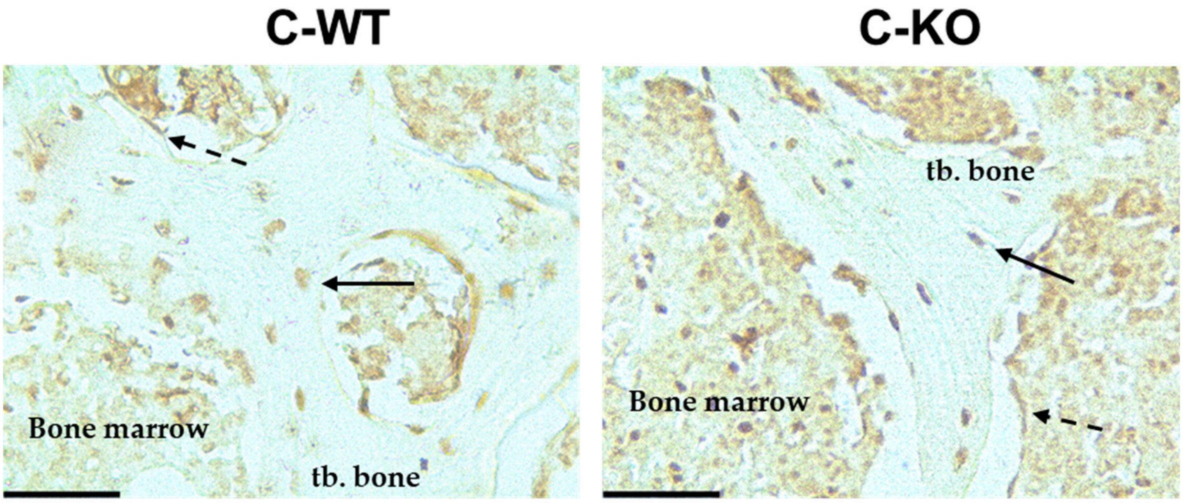

Figure S4. Representative IHC images of MEF2C staining from C-WT and C-KO mice. Solid arrows indicate osteocytes and dashed arrows indicate osteoblasts. Bone sections from four C-WT and four C-KO mice were stained for comparison.

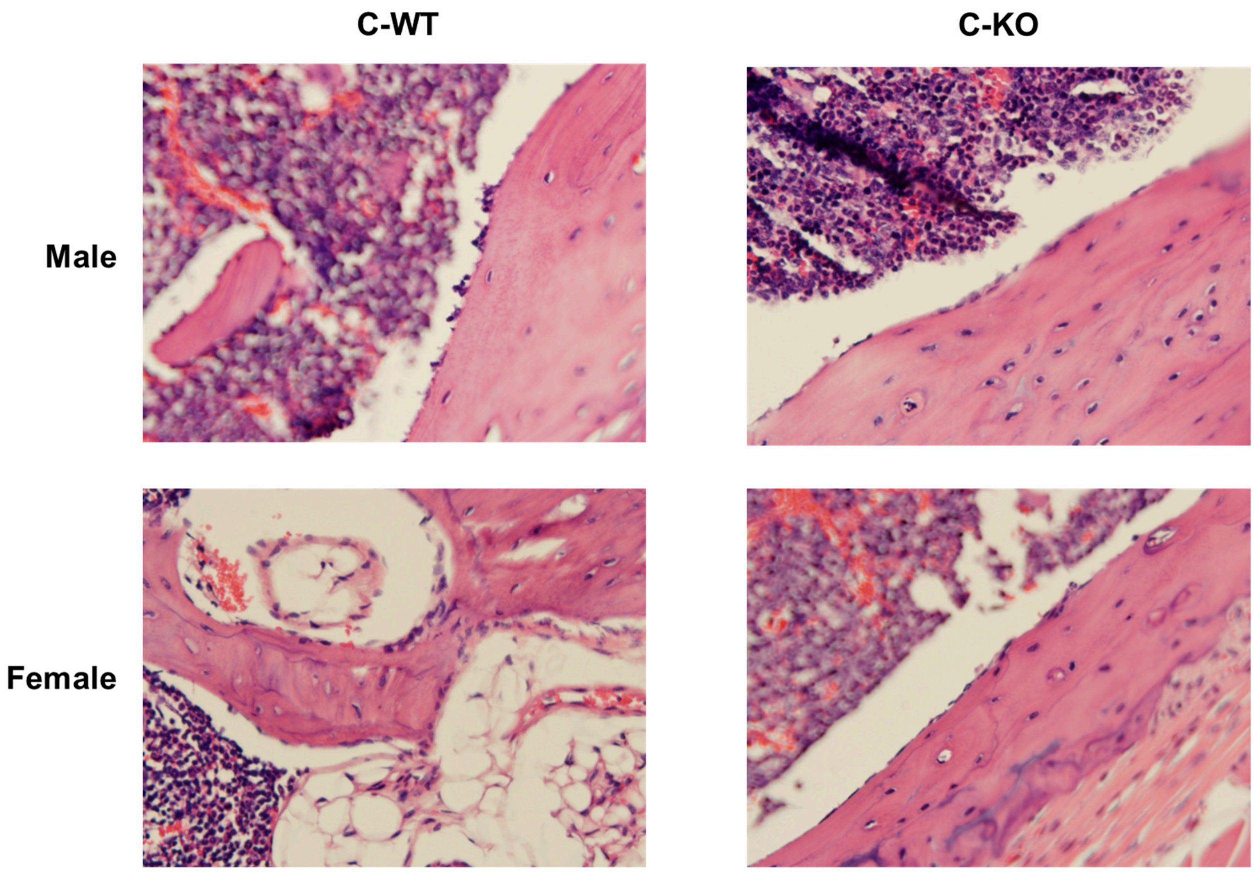

Figure S5. Representative H and E-stained sections from male as well as female C-WT and C-KO mice.
